# Chasing the FoxO in Metabolic Disorders: Novel Considerations for Oxidative Stress, Programmed Cell Death, Wnt, and the Gut Microbiome

**DOI:** 10.3390/antiox15070895

**Published:** 2026-07-20

**Authors:** Kenneth Maiese

**Affiliations:** Cellular and Molecular Signaling, New York, NY 10022, USA; wntin75@yahoo.com

**Keywords:** artificial intelligence, diabetes mellitus, glucagon-like peptide-1 receptor agonist, gut microbiome, mammalian forkhead transcription factors, mechanistic target of rapamycin, programmed cell death, silent mating type information regulation 2 homolog 1, Wnt, Wnt1 inducible signaling pathway protein 1

## Abstract

Lifespan is increasing throughout the world leading to a rise in non-communicable diseases in the global population that impacts over 800 million individuals with metabolic disorders, such as diabetes mellitus. Metabolic disease presents a significant challenge for clinical care since multi-organ disease progression ensues despite a broad array of treatment protocols. The pursuit of innovative strategies with mammalian forkhead transcription factors of the “O” class (FoxOs) and intimately related pathways of aging, cellular senescence, telomere integrity, oxidative stress, programmed cell death with apoptosis, autophagy, ferroptosis, pyroptosis, and cuproptosis, Wnt/β-catenin signaling, Wnt1 inducible signaling pathway protein 1, and the gut microbiome becomes vital to address the clinical hurdles of metabolic disorders. Platforms incorporating novel diagnostics with artificial intelligence and machine learning can further address the underlying mechanisms tied to FoxOs that include the mechanistic target of rapamycin, AMP activated protein kinase, silent mating type information regulation 2 homolog 1 (*S. cerevisiae*), and glucagon-like peptide-1 receptor agonists that can markedly influence biological outcomes. Given the premise that it is essential to comprehend the intimate relationship that FoxO signaling pathways hold, FoxOs offer an exciting and promising approach to address the clinical aspects of disease onset, progression, and treatment with metabolic disorders.

## 1. Introduction

Lifespan is increasing throughout the globe and this has led to a corresponding rise in non-communicable diseases (NCDs) [1,2]. NCDs include cancer, cardiac disease, respiratory disease, dementia, trauma, musculoskeletal disorders, renal disease, suicide, and diabetes mellitus (DM). NCDs lead to over 75 percent of deaths each year in the world, result in over 43 million deaths, with approximately half of these deaths occurring prior to age 70, and over 80 percent of the deaths occurring in low- and middle-income countries [3]. NCDs can have cyclic presentations, such as with respiratory disease or deaths related to substance abuse, illustrated by improved care with infections from severe acute respiratory syndrome coronavirus 2 (SARS-CoV-2) and coronavirus disease 2019 (COVID-19) as well as recent decreases in mortality observed with drug overdoses [4,5,6]. Yet, disorders associated with metabolic dysfunction including DM continue to impact a significant proportion of the global population.

**Figure 1:**
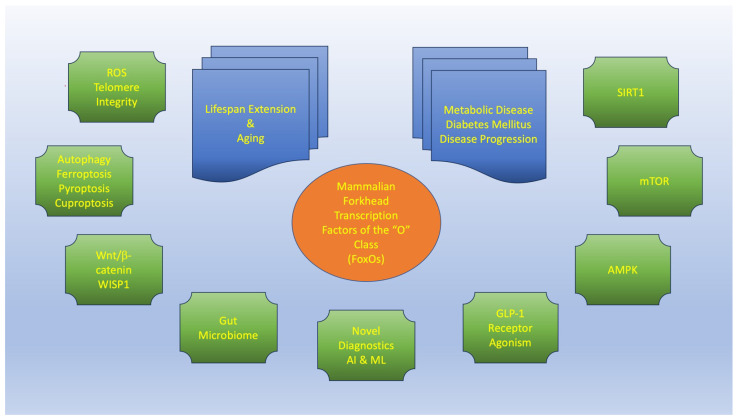
Mammalian forkhead transcription factors of the “O” class (FoxOs) offer innovative avenues to address metabolic disorders. With increased aging and lifespan, metabolic disease, such as diabetes mellitus, presents significant challenges with progressive disorders for individuals. Innovative strategies with mammalian forkhead transcription factors of the “O” class (FoxOs) and intimately related pathways of cellular senescence with telomere integrity, oxidative stress and reactive oxygen species (ROS) generation, programmed cell death with apoptosis, autophagy, ferroptosis, pyroptosis, and cuproptosis, Wnt/β-catenin signaling, Wnt1 inducible signaling pathway protein 1, and the gut microbiome are vital to address the clinical aspects of metabolic disorders. Platforms that employ novel diagnostics with artificial intelligence (AI) and machine learning (ML) can assist with the assessment of FoxO signaling and mutual pathways that include the mechanistic target of rapamycin (mTOR), AMP activated protein kinase (AMPK), silent mating type information regulation 2 homolog 1 (*S. cerevisiae*) (SIRT1), and glucagon-like peptide-1 (GLP-1) receptor agonists that can mediate both beneficial and detrimental biological outcomes.

The associated increase in lifespan with the global population also promotes the processes of advanced aging that can influence metabolic function (Table 1). Over the next 5 years, the age of the world’s population will continue to increase, with the expectation that almost a quarter of these individuals will live beyond the age of 60 [7,8]. During the course of the next 25 years, greater than 2 billion individuals will be over the age of 60 and almost 430 million individuals will reach the age of 80 or older [9,10]. Multiple factors can extend lifespan, which involve improved sanitation measures, reduced exposure to environmental toxins, increased public education, greater access to healthcare, early use of clinical diagnostics, and growth of public health policies [10,11]. With this aging of the population, metabolic disorders that include DM continue to increase in prevalence. As individuals reach greater than 65 years of age, almost 136 million people will suffer from DM, and by the year 2045, this will increase to over 275 million individuals [12]. Underlying the processes of aging that contribute to metabolic dysfunction are the onset and induction of cellular senescence. The processes leading to cellular senescence can promote inflammation, vascular disease, lipid metabolism dysfunction, pancreatic β-cell injury, alterations in mitochondrial dynamics, programmed cell death, and gut microbiota changes [13,14,15,16,17]. Cellular senescence is overseen by telomere (TL) function. TLs are positioned on the ends of chromosomes, consist of deoxyribonucleic acid (DNA) complexes, contain over 2000 repetitions of non-coding double-stranded DNA, and have guanine-rich single-stranded DNA ends with the sequence “TTAGGG”. Ultimately, TLs oversee cell replication and survival with the protein complexes CTC1-STN1-TEN1 (CST), shelterin, and telosome to modulate activity and integrity of TLs [18]. As a mechanism of protection for TL integrity, telomerase protein exists to protect against base pair loss in TLs since 25–200 base pairs are depleted during cell division. Telomerase adds tandem repeat ribonucleic acid (RNA) templates for the maintenance of TL base pair length. With aging and the initiation of cellular senescence, TL integrity and function are lost when fewer than 500 base pairs in TLs exist, with the onset of cellular energy impairments, generation of oxidative stress, loss of metabolic homeostasis, and initiation of disorders such as DM (Figure 1).

DM represents a significant challenge for clinical care. DM affects all systems throughout the body and can result in neurodegenerative disorders, renal failure, hepatic disease, cancer, psychiatric disorders, and musculoskeletal disease [19,20,21]. It is estimated that greater than 800 million individuals, an increase from 200 million people three decades prior, have DM, with over 2 million deaths annually from this disorder [3,18]. A 20 percent increase in mortality rates with DM has occurred over the prior 2.5 decades when compared to other disorders, such as respiratory disease, cancer, and cardiac disease. Almost 15 percent of individuals older than 18 years of age have DM, an increase since the year 1990 of 7 percent [3,22,23]. In addition, it is estimated that more than 40 percent of individuals in the United States (US) alone suffer with pre-diabetes, and have elevated fasting serum glucose and hemoglobin A1c levels, but remain without a diagnosis or care [24,25]. On a financial basis, patients with DM can incur costs of 22,000 United States dollars (USD) per year and reach an aggregate basis of 400 billion USD [7,26]. Additional costs that involve functional loss with disability, occupational loss, home care, and institutional treatment result in charges that are 800 billion USD per year and greater than 2 percent of the US Gross Domestic Product [27,28].

Multiple factors can contribute to the onset of DM, which involve alcohol and tobacco consumption, SARS-CoV-2 infection, elevated serum cholesterol levels, socioeconomic status, lower education level, hypertension, decreased physical activity, and obesity [16,29,30,31]. Given challenges with poor care compliance, the education level may be an overlooked consideration since only 7 percent of individuals with DM have greater than a high school education level and 13 percent of individuals with DM have less than a high school education level. These observations suggest that new care therapies with real-time glucose monitoring may be helpful [32]. Furthermore, complications with obesity can lead to inflammation, stem cell loss, glucose intolerance, insulin resistance, progressive aging processes, mitochondrial dysfunction, susceptibility to infection, such as with COVID-19, and oxidative stress [33,34,35,36].

## 2. Mammalian Forkhead Transcription Factors of the “O” Class (FoxOs) as an Innovative Pathway to Pursue Metabolic Disorders

With the global increase in lifespan and aging, DM can affect approximately 15 percent of the individuals under the age of 70, and almost another 40 percent of people may suffer from metabolic disorders without a current diagnosis [3,22,23,24,25]. Yet metabolic disorders such as DM remain a significant clinical challenge, with continued multi-system disease progression despite therapies involving exercise plans, hypoglycemic treatments, weight management, and nutritional guidance. As a result, innovative disease care approaches are highly warranted [10,29,37,38,39]. Mammalian forkhead transcription factors of the “O” class (FoxOs) represent such an approach and have a vital role in the pathways of cellular metabolism, oxidative stress, reproduction, and programmed cell death pathways [21,40,41,42,43]. Following the discovery of the *Drosophila melanogaster* gene *forkhead*, greater than 100 genes and 19 human subgroups with *FOXA* to *FOXS* have been described. Additional terminology for forkhead proteins are forkhead in rhabdomyosarcoma (FKHR) (FOXO1), FKHRL1 (forkhead in rhabdomyosarcoma like protein 1) (FOXO3a), Forkhead RElated ACtivator (FREAC)-1 and -2, the *Drosophila* gene *fork head* (*fkh*), and the acute leukemia fusion gene located in chromosome X (*AFX*) (*FOXO4*). Mammalian FOXO proteins of the “O” class include FOXO1, FOXO3, FOXO4, and FOXO6, and their activities are conserved among several species, which include *Caenorhabditis elegans*, *Drosophila melanogaster,* and mammals [44]. Mammalian FOXO proteins maintain a butterfly-like appearance on X-ray crystallography and nuclear magnetic resonance [44], and the forkhead box (FOX) family of genes has a conserved forkhead domain noted as a “winged helix” [45,46].

## 3. Oxidative Stress, Programmed Cell Death, and FoxOs

FoxOs are intimately tied to the pathways of oxidative stress and programmed cell death. Oxidative stress is a critical component of cellular metabolic dysfunction and occurs through the generation of reactive oxygen species (ROS) that include peroxynitrite, hydrogen peroxide, singlet oxygen, nitric oxide, and superoxide free radicals [47,48]. Oxidative stress during metabolic dysfunction can affect multiple systems of the body to lead to cardiac injury [49,50], circadian rhythm dysfunction [51,52,53], endothelial cell injury [50,54], stem cell impairment [55,56], loss of mitochondrial dynamics [40,57], inflammation [16,58,59], neuronal, astrocytic, and microglial demise [60,61,62,63], demyelination [64,65,66], cellular senescence with loss of TL integrity [18,67], and reduction in growth factor cellular protection [41,47,68]. The effects of DM and oxidative stress on systems such as growth factor expression exemplify the ability ROS to alter normal physiological processes [59]. With the reduction in insulin like growth factor-1 levels during DM, mitochondrial function is lost, and superoxide dismutase enzyme activity that can limit ROS under normal conditions becomes diminished [69,70]. In a similar manner, the growth factor erythropoietin (EPO) can be affected through pathways of oxidative stress to limit the ability of EPO to protect against programmed cell death [71]. EPO cellular protection can occur through the upstream modulation of hypoxia-inducible factor-1α [72,73,74,75,76] as well as through pathways of protein kinase B (Akt) and the mechanistic target of rapamycin (mTOR) to increase cellular survival and maintain metabolic homeostasis [38,77,78,79]. Through pathways of Akt and mTOR, EPO can promote microvessel integrity, maintain glucose homeostasis, improve cardiac function, foster angiogenesis, and assist with the re-myelination of nerves [25,80,81,82,83] (Table 1).

Cellular demise through oxidative stress is not a linear process and is dependent upon several factors (Table 2). For example, the detrimental effects of oxidative stress can be highly reliant upon tissue specificity, cellular energy pathways involving the coenzyme ß-nicotinamide adenine dinucleotide (NAD^+^), gender, and mechanisms involving inflammation [48,84,85,86,87]. Furthermore, antioxidant systems in the body can oversee and temper the effects of ROS through entities that involve superoxide dismutase enzyme, glutathione peroxidase, catalase, and the vitamins K, B, C, D, and E [10,87,88,89,90,91]. During periods when antioxidant systems are minimized, oxidative stress ensues as a result of imbalances in the ratio of antioxidants and oxidants. If the appropriate ratio is maintained, then beneficial effects can result, such as with fostering neural stem cell survival and plasticity [55,92,93], limiting endothelial cell injury [48,54,94], protecting neuronal and musculoskeletal cells [95,96,97], and enhancing hepatic cell survival [98,99,100,101] (Figure 1).

Cellular metabolic dysfunction and oxidative stress are influenced by the pathways of programmed cell death, which include apoptosis, autophagy, ferroptosis, pyroptosis, and cuproptosis (Table 1). In the initial stages of apoptosis, injured cells, such as in the nervous system, have externalization of membrane phosphatidylserine (PS) residues that attracts microglial cells to target and remove injured cells with exposed membrane PS residues [7,61,102,103]. Microglia oversee the initial stages of apoptotic cell injury during oxidative stress to engulf cells that appear to be non-functional [10,62,104,105]. For these reasons, microglial cell survival is a vital factor during programmed cell death, and promotion of microglial cell integrity can occur by triggering receptor expressed on myeloid cells 2 (TREM2) to modulate microglial cell polarization, reduce inflammation, and limit oxidative stress [18,104,106,107,108]. Microglial cells also can be protective to enhance cell survival during metabolic dysfunction and oxidative stress by increasing brain autophagic flux to promote autophagy-related genes Atg6, Atg7, and Atg12 transcription, which can control agitation, social avoidance, and mood disorders [109]. Microglia can also reduce Aβ toxicity in disorders such as Alzheimer’s disease (AD) [110,111,112,113,114], which involves the transient receptor potential cation channel subfamily V member 2 (TRPV2) and phagocytosis of Aβ deposition [101,108,115] as well as other pathways involving innate immunity and mTOR [61,116,117]. The TRPV1 family receptors may also function in AD with co-morbidities of DM to reduce oxidative stress and modulate tau phosphorylation [7,118,119,120]. With the multiple roles that microglia can play during apoptosis, it is vital to note that prior to the induction of the second phase of apoptosis, the initial phase can be a reversible process. Reversal of membrane PS externalization can occur on cells that are recovering from injury or are assisted to recover through therapeutic means. This internalization of membrane PS residues prevents phagocytosis from microglia, allowing normal function of the recovering cells [10,121]. If this reversal process does not occur, then the final phase of apoptotic cell death can ensue with mitochondrial membrane depolarization, release of cytochrome c, caspase activity, and degradation of DNA [101,122,123,124].

Autophagy is used by cells to recycle cellular components and cytoplasmic organelles for use in the future and can be an essential component to maintain cellular homeostasis [7,125,126]. Autophagy has three subtypes that include macroautophagy, microautophagy, and chaperone-mediated autophagy. Macroautophagy is the most common form of autophagy described and combines cytoplasmic proteins and cellular organelles into autophagosomes for lysosomal degradation [115,127]. Microautophagy recycles cellular organelles though lysosomes with the invagination of lysosome membranes, and chaperone-mediated autophagy relies upon lysosomes for organelle degradation with “protein chaperones” as the transfer mechanism [10,128,129,130,131]. Dysregulation of autophagy pathways, such as during metabolic instability with obesity [125], can promote loss of metabolic homeostasis with DM and lead to cognitive loss with AD [132]. Activation of autophagy in cognitive disease can limit brain Aβ deposition, remove tau accumulation, reduce oxidative stress, and slow processes associated with aging [61,115,133,134,135,136]. Induction of autophagy can decrease autophagosome accumulation to limit toxicity of Aβ [137], promote Aβ clearance [138], decrease ischemic brain injury during DM [74], limit cognitive loss during DM [139], and function to modulate tissue repair pathways [56]. If autophagy activation is lost, memory impairment, aging, and Aβ and tau accumulation can result [2,140]. Alterations in serum glucose changes during metabolic instability can lead to the loss of autophagy flux control and the activation of inflammatory pathways with microglia [141,142]. Resolution of diabetic retinopathy is dependent upon autophagy activation [136], autophagy can reduce insulin resistance through growth factors such as EPO [25,80], and autophagy is necessary in disorders such as multiple sclerosis with cognitive loss to reduce the release of cytokines, promote oligodendrocyte development and myelination, and oversee microglial inflammatory activity [65,66,143]. Autophagy also assists with β-cell proliferation in the pancreas [144], mitochondrial dynamics and mitophagic flux [17,122], reduction in insulin resistance during high serum lipid administration in obesity models of autophagy *Atg7* gene deletion [145], and nutritional benefits with DM that include oversight of flavonoids, fatty acids, gut microbiota, and physical exercise to promote metabolic homeostasis [17,146,147].

Although autophagy can be beneficial at times, modulation of autophagy flux levels can be crucial to achieve desired biological outcomes with metabolic disease (Table 2). In the presence of advanced glycation end products (AGEs) during DM, autophagy activation can lead to oxidative stress, endoplasmic reticulum stress, DM retinopathy, and atherosclerosis [2,148,149,150]. Heightened induction of autophagy can result in stem cell loss [8,114], Aβ and tau toxicity [151,152,153], oxidative stress [154,155], susceptibility to infection, such as with SARS-CoV-2, and increased risk of death [66,156], neuronal cell injury [140], behavioral disorders with depression [146], and circadian rhythm disruption during DM [21,157,158,159]. Regulation of autophagy activity can occur through mTOR, which has a reciprocal relationship with autophagy. Activation of mTOR, also termed the mammalian target of rapamycin and the FK506-binding protein 12-rapamycin complex-associated protein 1 [7,47,160], can function to modulate autophagy flux levels [10,127,161]. mTOR can work in concert to balance autophagy activity to control cellular metabolism with AMP activated protein kinase (AMPK) [10,29,162,163], limit oxidative stress [7,164,165], oversee lysosome regulation and accumulation of autophagosomes [137,166], prevent atherosclerotic plaque instability [167], maintain TL integrity [168], modulate astrocytic cell activity [63], and minimize viral susceptibility and infection with SARS-CoV-2 and COVID-19 [6,169,170]. With cellular protection by growth factors such as brain-derived neurotrophic factor, mTOR activity with control of autophagy flux can reduce Aβ toxicity [171], limit inflammation [172], and increase neuronal survival [165]. To a similar degree, EPO relies upon mTOR to reduce insulin resistance [78], promote integrity of TLs [168], foster proliferation of cells [160], alleviate myelopathy [81], and limit oxidative stress [59,173,174].

Ferroptosis, pyroptosis, and cuproptosis are additional pathways of programmed cell death that can function independently or in concert with autophagy and apoptosis during metabolic disorders. Ferroptosis involves iron accumulation in cells, which leads to lipid peroxidation, generation of oxidative stress, and the loss of glutathione homeostasis [2,175]. Ferroptosis can affect multiple systems and lead to neuronal loss with epilepsy [176], traumatic brain injury [177], neurodegeneration [104], demyelination [65,66], peripheral nerve injury [178], and endoplasmic reticulum stress [179]. Renal disease, especially during DM, can be mediated through ferroptosis [175,180]. Ferroptosis can result in cardiomyocyte injury [16,181,182], cellular energy stress with mitochondrial dysfunction [21,183], and inflammation [184,185,186]. Ferroptosis also may be a target for tumorigenesis [187,188] and a biomarker for recurrent miscarriage [189]. Pyroptosis programmed cell death involves inflammatory pathways that ultimately can result in cytokine release and caspase activation [2,186,190]. The inflammasome family of nucleotide-binding oligomerization domain-like receptor and leucine-rich repeat-containing receptors (NLRs) containing NLRP1, NLRP3, NLRP6, and NLRC4 are components of pyroptosis programmed cell death that employ pattern recognition receptors responding to damage-associated molecular pattern (DAMP) and pathogen-associated molecular pattern (PAMP) molecules controlled through the inflammasome, also termed the pyroptosome, with the NLRs gasdermin proteins [191,192,193]. Once pyroptosis is activated, cytokine induction and caspase activity occur with caspase 1, caspase 4, and caspase 5 [194]. Pyroptosis is involved with SARS-CoV-2 and COVID-19 infection [2,195], inflammation during DM wound healing [196,197], endothelial cell injury [198], metabolic pathways with DM that involve non-coding RNAs [199], renal injury during DM [191], cognitive impairment and neuronal loss with obstructive sleep apnea [200], cancer pathways [194], cardiac disease with DM [182,197,201], and nervous system injury with tau [202]. Cuproptosis is a distinct form of programmed cell death that involves the abnormal accumulation of mitochondrial copper, which results in cellular stress. Copper ions bind to lipoylated proteins involved in the tricarboxylic acid cycle, which leads to protein aggregation, destabilization of the cell, and mitochondrial dysfunction [104,186]. Through mitochondrial impairment, cuproptosis results in loss of glucose homeostasis and is associated with DM cardiomyopathy [203], oxidative stress [64,204], motoneuron impairment [104], cognitive loss with microglial inflammation in models of AD [62], and insulin resistance [205] (Figure 1).

In experimental studies, exposure to manganese that alters cellular energy pathways can result in neurotoxicity through FoxO-mediated oxidative stress pathways [206]. Furthermore, FoxO3a activity can promote oxidative stress and stem cell dysfunction with osteoblastic differentiation [207], FoxO3 can generate gasdermin D activity to lead to programmed cell death with pyroptosis, autophagy, and cell demise [194,208], and FoxOs can result in reproductive dysfunction [43,209]. In the brain, FoxOs can contribute to oxidative stress, inflammation, and apoptotic cell death during cerebral hemorrhage [210]. FoxO3a can work in concert with cuproptosis to suppress Wnt signaling pathways of β-catenin, leading to mitochondrial dysfunction and cellular energy depletion [211].

It is important to recognize in some scenarios that a critical level of FoxO activity is required for cellular viability against oxidative stress and pathways of programmed cell death (Figure 1). During periods of oxidative stress, exposure in models of osteoarthritis and chondrocyte cell survival, FoxO1 and FoxO3 activity is necessary to provide oxidative stress resistance and tissue homeostasis for improved cellular survival [212] (Table 2). Restoration of depressed FoxO3a levels may be necessary to promote cytoprotection during cerebral ischemia and oxidative stress [213]. Cytoprotection by FoxOs may be mediated in part through the promotion of DNA repair mechanisms since FoxOs can assist with glutamine synthetase expression in astrocytes as a protective mechanism to reduce neuronal excitotoxic injury and cognitive loss in disorders such as AD [214]. In addition, with a regulated activity of FoxO1 and FoxO3, antioxidant catalase and superoxide dismutase can have increased expression to limit damage in cardiac [215] and brain [216] tissues. During periods of programmed cell death with autophagy and ferroptosis, FoxOs, such as Foxo3a and FoxO4, can limit oxidative stress and prevent the induction of programmed cell death [21,181,183]. FoxOs can function in concert with energy pathways that involve AMPK to limit oxidative stress and maintain cellular energy homeostasis [183,217,218]. In studies examining oxidized low-density lipoproteins and oxidative stress, treatment with humanized IgG1 antibody can resolve oxidative stress through FoxO1 activation and SIRT1 expression [219].

## 4. FoxOs and Metabolic Pathways

In regard to cellular metabolism and mechanisms of action, FoxO proteins are homologous to the transcription factor DAuer Formation-16 (DAF-16) in *Caenorhabditis elegans*, which oversees regulation of the cell cycle, insulin signaling, and lifespan extension [44,220,221]. As transcription factors, FoxO proteins bind to DNA through the FoxO-recognized element in the C-terminal basic region of the forkhead DNA binding domain. This domain has 14 protein-DNA contacts, which controls gene expression of targets in the α-helix H3 recognition region. FoxOs are regulated through epigenetic and post-translation protein modifications that can involve protein acetylation, protein phosphorylation, ubiquitylation, nuclear compartmentalization of FoxOs, and electrostatic charge changes [41,66,163,222]. In addition, both Akt and mTOR are tightly linked to FoxO proteins. FoxO proteins can be phosphorylated by Akt, lead to mTOR activation, and prevent nuclear translocation of FoxO through the association of FoxO with cytoplasmic 14-3-3 proteins that prevent activation of caspase pathways and apoptosis induction [61,75,223]. Alternate routes also exist to modulate programmed cell death, metabolism, and cellular energy stores with FoxOs. For example, agents such as nicotinamide, which can influence cellular energy and metabolism, can activate Akt to promote FoxO3a protein integrity, prevent FoxO3a proteolysis, and block the existence of “pro-apoptotic” amino-terminal (Nt) fragments that independently can result in apoptotic cell death [16,224,225,226]. Ubiquitination and the degradation of FoxO proteins can be mediated via the silent mating type information regulation 2 homolog 1 (*S. cerevisiae*) (SIRT1) [42,227,228,229,230]. SIRT1 is a histone deacetylase that controls transcription of DNA through acetyl group transfer from ε-N-acetyl lysine amino acids to histones of DNA. SIRT1 is expressed in tissues throughout the body and can be found in the spleen, liver, pancreas, heart, adipose tissue, skeletal muscle, and brain [77,123,225]. SIRT1 oversees nicotinamide phosphoribosyl-transferase (NAMPT), which is necessary for nicotinamide adenine dinucleotide (NAD^+^) production, a substrate for SIRT1 [10,231]. SIRT1 can control the SIRT1-FoxO axis to protect against Aβ and tau [163,232,233], prevent neuronal developmental effects [229], block hippocampal neuronal demise [234], and limit oxidative stress during loss of metabolic homeostasis [223,235]. Interestingly, FoxOs can be a transcriptional activator of SIRT1 and increase SIRT1 expression through the SIRT1 promoter region that contains a cluster of five putative FoxO core binding repeat motifs (5 x insulin receptor substrate (IRS-1)) and a forkhead-like consensus-binding site (FKHD-L) [236] (Table 2). SIRT1 also leads to FoxO-driven SIRT1 autotranscription through the activation and deacetylation of FoxOs [236,237]. SIRT1, as a histone deacetylase, deacetylates FoxO to lessen the ability to promote apoptotic cell death [238]. FoxOs are acetylated by histone acetyltransferases that include p300, the CREB-binding protein (CBP), and the CBP-associated factor and have reduced activity since acetylation of FoxO lysine residues can limit FoxO proteins to bind to DNA [239]. Akt can promote ubiquitination and degradation of FoxOs through the 26S proteasome of FoxO proteins [223,240].

During metabolic dysfunction, FoxOs can have a critical influence over cellular survival (Table 1). In high-glucose exposure studies, FoxO1 activity can be increased with a reduction in activation of SIRT1, resulting in endothelial cell senescence and dysfunction [241]. FoxO activity in the presence of reduced SIRT1 activity impairs wound healing during DM by reducing the necessary numbers of fibroblasts, macrophages, and mast cells to the wound healing site [242]. In the presence of FoxO activity that involves FoxO6, hepatic gluconeogenesis is increased, insulin sensitivity is lost, insulin resistance is enhanced, and inflammation with macrophage recruitment is elevated [243]. During hyperglycemia, FoxO3a can impair endothelial progenitor cell function, disrupt blood flow, and lead to ischemic injury during DM [235,244]. FoxO1 elevations and high-mobility group A1, a chromatin-binding protein, may lead to spatial memory loss and hippocampal dysfunction in models of AD and DM [245]. In experimental models for small-for-gestational-age offspring, FoxO1 can contribute to metabolic dysfunction and the loss of neuroprogenitor cells [246]. FoxO1 may alter pancreatic β-cell biology by interfering with thioredoxin-interacting protein that is necessary for pancreatic β-cell survival [247].

## 5. Wnt Signaling, WISP1, the Gut Microbiome, and Novel Diagnostics with Artificial Intelligence

FoxOs are closely associated with the pathways of Wnt signaling and Wnt1 inducible signaling pathway protein 1 (WISP1) (Table 1). Modulation of FoxOs through direct and autoregulatory mechanisms by Wnt and WISP1 oversee cellular survival and cellular energy pathways. Wnt signaling proteins are cysteine-rich glycosylated proteins that are part of the *wingless* pathway, include β-catenin, and oversee cellular development, metabolism, survival, and tumorigenesis [7,49,248]. During metabolic dysfunction, Wnt signaling and the β-catenin pathway can limit vascular and microglial injury with EPO, which oversees FoxO3a regulation [79,249]. Wnt signaling proteins may offer an early diagnostic tool for neuropathy, cerebral ischemia, cognitive performance, and renal disease during DM [250,251,252,253]. Activation of the Wnt/β-catenin pathway is necessary for osteogenic differentiation that may prevent osteoporosis through FoxO3a inhibition, which can occur with metabolic dysfunction [207,254]. In models of experimental DM, Wnt/β-catenin signaling can function through EPO and FoxO3a inhibition to maintain vascular integrity [255]. Wnt/β-catenin signaling can reduce oxidative stress and Aβ injury in microglia [117], prevent mitochondrial injury and ischemic neuronal demise in the brain [248,256], potentially assist with wounds during DM [257], limit hepatic cell injury through blockade of FoxO3a [258], and block renal mesangial cell demise during DM [259,260].

However, Wnt signaling can have a dual role in cytoprotection, suggesting a need for vital oversight of a close biological control for Wnt signaling to foster desired outcomes [7,23,251,261]. Reduction in vascular calcification during DM may require SIRT1 activity with inhibition of the Wnt/β-catenin pathway [262]. Cardiac toxicity and cognitive loss can be fostered by the activation of the Wnt pathway [49,263,264]. With aging and effects of DM on osteogenesis, limits on the activation of Wnt signaling and FoxOs with increased SIRT1 expression are required to maintain and increase bone mass [265]. In models of DM retinopathy, reduction in Wnt signaling can prevent inflammation and vascular leakage [22,266,267,268,269]. Neuropathic pain can be mediated through Wnt signaling and FoxO1 [270] and neuronal excitability may require modulation of Wnt signaling with SIRT1 upregulation [176]. In regard to tumorigenesis, reduction in Wnt signaling, such as through FoxO1 and FoxO3a, can block malignant phenotypes [271,272]. Wnt signaling also may have a feedback loop on FoxOs and can block FoxO transcription, such as with FoxO3a [273] (Table 2).

WISP1, also termed CCN4, is a member of the CCN family of six secreted extracellular matrix proteins that can modulate programmed cell death, metabolism, oxidative stress, stem cell proliferation, and cellular survival, which also functions with FoxO proteins [7,274]. WISP1 is a downstream target of Wnt signaling and is present in the pancreas, small intestine, cardiovascular system, nervous system, spleen, epithelium, placenta, pulmonary system, ovaries, and musculoskeletal system [7,275,276]. In metabolic disease, WISP1 levels and other WISP family members are increased during obesity, gestational DM, and adult DM and may be a biomarker for inflammation [277,278,279,280,281], cellular remodeling [282], and adipose tissue dysfunction [33,283]. WISP1 may be associated with glucose and lipid metabolism during obesity to decrease insulin resistance [284]. During metabolic disease, WISP1 can prevent nuclear translocation of FoxO3a and increase adipocyte survival as well as maintain glucose homeostasis [274]. WISP1 modulates cellular metabolism through various pathways, which involve mTOR, AMPK, FoxO, and autophagy, and can autoregulate its own expression through β-catenin and programmed cell death pathways [285] (Table 2). WISP1 can block Aβ toxicity through mTOR activation and related pathways of the proline-rich Akt substrate 40 kDa (PRAS40) [286]. WISP1 can oversee AMPK phosphorylation by limiting tuberous sclerosis 2 (TSC2) phosphorylation at serine^1387^, a target of AMPK, and increasing TSC2 phosphorylation at threonine^1462^, a target of Akt [7,134,287,288]. Through AMPK, WISP1 can control the cellular lifespan and cellular senescence [289]. WISP1 can prevent mitochondrial membrane depolarization and inhibit deacetylation of FoxO3a to block apoptotic caspase activation and promote SIRT1 protection of cells [223,290,291]. Yet, similar to Wnt signaling, excessive activity of WISP1 may be detrimental and lead to an increased risk of cerebral infarction during DM [252]. WISP1 also can activate Akt and mTOR to increase cytokine release, such as interleukin-6, which promotes inflammation [292,293,294]. WISP1 can mediate inflammation through macrophage migration inhibitory factor expression, which can involve pyroptosis pathways to foster cell injury [295] (Figure 1).

Interestingly, the gut microbiome plays an integral role in the regulation of cellular metabolism, which involves FoxOs. The gut microbiome, also known as the gut flora or the gut microbiota, includes bacteria, fungi, viruses, and archaea in the gastrointestinal tract [10,163,296]. With over 100 billion bacteria, the gut microbiome is an extension of the endocrine system and generates short-chain fatty acids (SCFAs) as an energy source and vitamins such as vitamin B [10,297,298,299,300]. The gut microbiome offers a therapeutic avenue to treat oxidative stress and metabolic dysfunction through the alteration of Lactobacillus, Bacteroidota, and Firmicutes species, which can alter mTOR and AMPK activities to reduce inflammation and improve glucolipid metabolism [301,302,303,304]. The gut microbiome also is dependent upon SIRT1 activity to regulate glucose and hepatic lipid homeostasis through gut microbiome Firmicutes and Bacteroidetes [305], limit oxidative stress and aging processes [301], maintain vascular integrity [306], and modulate indole metabolites, such as 3-indollepropionic acid, to limit Aβ and tau toxicity [163]. Clearance of the Aβ and tau aggregates employs gut microbiome indole metabolites with the FoxO3a signaling pathway [163]. Forkhead signaling also can work with the Wnt/β-catenin pathway to activate a mesenchymal and epithelial cross-talk communication in the gut for extracellular proteoglycans, which can function as co-receptors for Wnt signaling [307]. FoxOs, such as FoxO1 and FoxO3, also control gut homeostasis through the regulation of intestinal mucous secretion in goblet cells, which can impact SCFA-producing bacteria [308] and oversee macrophage polarization through SCFAs and FoxO3 [309].

SCFA generation in the gut also results in the production of glucagon-like peptide-1 (GLP-1) [310] (Table 1). GLP-1 receptor agonism pathways are clinically relevant for the treatment of DM and obesity with current US Food and Drug Administration approval [10,101,311,312]. In addition, GLP-1 receptor agonists are applicable for nonalcoholic fatty liver disease, also known as metabolic dysfunction-associated steatotic liver disease [101,313]. GLP-1 receptor agonism can have positive benefits for insulin resistance reduction [311], maintenance of mitochondrial integrity [314,315], and preservation of glucose homeostasis through TRPV1 receptor activation [7,101,316,317]. In regard to FoxOs, GLP-1 receptor agonism can lead to Akt and mTOR activation with FoxO phosphorylation and inhibition to promote pancreatic β-cell growth and regeneration. GLP-1 receptor agonism prevents nuclear translocation of FoxO1 and, as a result, proliferation of pancreatic β-cells is promoted, which can improve glucose homeostasis through GLP-1 receptor agonism and FoxO1 inhibition [318]. Yet the relationship between FoxOs and GLP-1 receptor agonists can have alternative biological outcomes if autophagy induction ensues. In toxic environments to pancreatic β-cells, GLP-1 receptor agonism can provide cellular protection to pancreatic β-cells through the induction of autophagy pathways, which is mediated by FoxO1 activation [319]. These pathways of GLP-1 receptor agonism that involve FoxOs are reliant upon the modulation of mTOR pathways and autophagy. Activation of mTOR with GLP-1 receptor agonism can foster glucose homeostasis and pancreatic β-cell proliferation [7,146,320] as well as block cholesterol-induced cell death [312]. However, GLP-1 receptor agonism also can inhibit mTOR activity with the induction of autophagy to limit inflammation, angiogenesis, and retinal injury during DM retinopathy [136] and to protect pancreatic β-cells with FoxO1 activation [319] (Table 2). Careful oversight of mTOR and autophagy activities with GLP-1 receptor agonism and FoxOs is vital for seeking desired clinical outcomes and to limit potential toxic effects with GLP-1 receptor agonism, such as pancreatitis, depression, vomiting, nausea, anterior ischemic optic neuropathy, alopecia, and diarrhea [101,321,322,323,324] (Figure 1).

The implementation of novel diagnostic techniques with the incorporation of artificial intelligence (AI) and machine learning (ML) programs is a critical consideration to assist with the early detection of metabolic disorders and the integration of innovative studies that consider FoxOs with aging, cellular senescence, oxidative stress, programmed cell death, Wnt signaling, and WISP1. The development of new diagnostics that can detect endothelial cell dysfunction that is related to DM, atherosclerosis, and hypertension with non-coding RNAs, such as circular RNAs, offers the potential to identify individuals with metabolic dysfunction at early disease stages [14,21]. Biomarkers found in body fluids that include serum, plasma, urine, bile, and exosomes may detect the progression of metabolic disease with DM, with new levels of sensitivity [7,325]. Early cellular energy deprivation states may be recognized at initial clinical disease stages with the monitoring of soluble leptin receptors [326]. Analysis of genetic variants of growth factor entities, such as EPO, in patients with DM also may identify those individuals associated with the risk of greatest mortality [327]. The detection of epigenetic rearrangements, gut microbiome composition changes, and increased processing of senescent cells offer the ability to detect, follow, and treat metabolic disorders such as DM [328]. Neurofilament light chain levels can function as either serum or plasma biomarkers to detect early neuronal cell loss, neuropathies, memory loss, and injury in the nervous system during DM [7,329]. Early onset of cognitive loss may be signaled by the depression of FoxO1 levels in the brain cortex in experimental models of AD, suggesting that biomarkers of FoxOs may be implemented for the early detection of disorders associated with metabolic dysfunction [330].

The information gained through the use of clinical diagnostics can be vast and involve non-homogenous data that require AI and ML techniques to adequately assess this information (Figure 1). AI and ML are platforms that can assess non-homogeneous data, which include genetic information, cellular pathways, pathological tissue, imaging, and liquid biopsies, to yield clinical predictive analysis that cannot be obtained from the singular use of standard statistical methods [10,331,332,333,334,335,336]. AI is being implemented to assess Wnt signaling in the development of DM microvascular complications with retinopathy, nephropathy, and neuropathy, and macrovascular complications that can involve coronary artery disease and peripheral artery disease to identify new therapeutic strategies [268]. For the development of new treatment regimens, AI and ML are being used to assess cardiometabolic disorders with the alignment of treatment to biological rhythms [337,338]. ML methods have identified candidate genes for neuropathic pain, a frequent problem with DM, to demonstrate that target genes rely upon FoxO1 to oversee neuropathic pain through Wnt signaling pathways [270]. Related disorders with metabolic disease that involve the pulmonary system have also been targeted by researchers, who have used ML to identify FoxO deficiency with increased risk of air space enlargement and chronic obstructive pulmonary disease [339]. Studies that use ML and several parameters to assess patients with DM, which include serum glucose, triglycerides, high-density lipoproteins, and WISP signaling, have identified WISP as a potential biomarker for the development of DM [33]. These studies with AI and ML highlight the integration of the multiple metabolic pathways with FoxOs that can determine the onset, progression, and treatment regimen for disorders such as DM.

## 6. Data Sources

Data sources were based on a systematic literature search using PubMed, Scopus, Web of Science, and ScienceDirect databases from January 2021 to June 2026. The search terms included “forkhead transcription factors”, “mechanistic target of rapamycin”, “silent mating type information regulation 2 homolog 1”, “artificial intelligence”, “glucagon-like peptide-1 receptor agonist”, “metabolism”, “diabetes mellitus”, “programmed cell death”, “gut microbiome” “oxidative stress”, “Wnt”, and “ Wnt1 inducible signaling pathway protein 1” with Boolean operators (AND, OR) to focus the search strategy. Peer-reviewed original research and review papers including the citations in the review papers were included, while non-peer reviewed work, duplicate studies, unrelated studies, and abstracts or meeting presentations with incomplete information were excluded.

## 7. Conclusions and Future Perspectives

Lifespan continues to increase throughout the world, with a corresponding rise in NCDs and a specific expansion in the prevalence of metabolic disorders such as DM. Metabolic disorders are a significant challenge for clinical care, affect all systems of the body, and lead to 2 million deaths annually. Although multiple factors can contribute to metabolic disorders, such as a lower education level, alcohol and tobacco consumption, elevated serum cholesterol levels, SARS-CoV-2 infection, socioeconomic status, hypertension, decreased physical activity, and obesity, clinical care remains with several hurdles to overcome since treatments with increased physical activity, weight management, hypoglycemic agent applications, and improved nutrition do not entirely halt disease progression. As a result, innovative avenues for new strategies to address metabolic disorders are warranted, which involve FoxOs and intimately related cellular pathways that oversee aging and cellular senescence, oxidative stress, programmed cell death with apoptosis, autophagy, ferroptosis, pyroptosis, cuproptosis, Wnt signaling, WISP1, and the gut microbiome. Closely tied to the examination of these vital pathways for metabolic disorders are pathways of mTOR, AMPK, SIRT1, and GLP-1 receptor agonism and the incorporation of the platforms of novel diagnostics, AI, and ML. The pathways with FoxOs offer great potential for new considerations to address metabolic disease, but must be carefully addressed since desired biological outcomes can be influenced by specific cellular environments and pathways. For example, the detrimental effects of oxidative stress can be highly reliant upon tissue specificity, cellular energy pathways involving NAD^+^, gender, and mechanisms involving inflammation such that ROS generation at times may foster beneficial effects with stem cell survival and endothelial function. Although autophagy induction has many beneficial merits, excessive activity of autophagy flux levels during metabolic disease can result in oxidative stress, stem cell loss, DM retinopathy, GLP-1 receptor agonist off-target effects, and circadian rhythm disruption, which necessitates the modulation of autophagy pathways by mTOR and AMPK. FoxOs also play a dual role dependent upon the cellular environment, such that FoxOs with a reduction in SIRT1 activity during metabolic disease can result in impaired wound healing, insulin resistance, and neuronal dysfunction. Yet, in other cellular environments, in the presence of a necessary level of FoxO activity, FoxOs can function with SIRT1 and AMPK to limit oxidative stress, increase cellular survival, maintain energy homeostasis, and promote antioxidant pathway activation. A level of FoxO activity also is required with autophagy induction to protect pancreatic β-cells through GLP-1 receptor agonism. Similarly, Wnt/β-catenin signaling and WISP1 can protect vascular integrity and stem cell differentiation and work with FoxOs to control gut microbiome homeostasis and SCFA production, but modulation of Wnt/β-catenin signaling and WISP1 are required through SIRT1 and FoxO pathways for bone mass protection, reduction in cardiac toxicity, and limitation of cytokine and pyroptosis pathways. Continued understanding of the intricate and complex function of FoxOs and the multiple shared cellular pathways connected to FoxOs is vital for the successful clinical translation of these innovative strategies for metabolic disorders, such as DM.

## Figures and Tables

**Table 1:** Highlights.

Chasing the FoxO in Metabolic Disorders: Novel Considerations for Oxidative Stress, Programmed Cell Death, Wnt, and the Gut Microbiome
The age of the world’s population will continue to increase with the expectation that almost a quarter of these individuals will live beyond the age of 60. As a result, non-communicable diseases have significantly risen, meaning metabolic dysfunction, which includes diabetes mellitus (DM), will impact a significant proportion of the global population.
DM affects all systems throughout the body, leading to neurodegenerative disorders, renal failure, hepatic disease, cancer, psychiatric disorders, and musculoskeletal disease and presents a significant challenge for clinical care since disease progression continues unabated.
Mammalian forkhead transcription factors of the “O” class (FoxOs) and intimately related pathways of aging, cellular senescence, telomere integrity, oxidative stress, programmed cell death with apoptosis, autophagy, ferroptosis, pyroptosis, cuproptosis, Wnt/β-catenin signaling, Wnt1 inducible signaling pathway protein 1, and the gut microbiome offer innovative avenues to address the clinical hurdles of metabolic disorders.
Platforms with novel diagnostics, artificial intelligence, and machines can further enhance clinical care with FoxO pathways to have biological oversight, with the mechanistic target of rapamycin, AMP activated protein kinase, silent mating type information regulation 2 homolog 1 (*S. cerevisiae*), and glucagon-like peptide-1 receptor agonists.
FoxO signaling pathways offer exciting and high yield potential for new considerations to address metabolic disease, but must be carefully addressed with the intimate association among complex pathways.
Depending upon the cellular environment, FoxO signaling and autophagy flux levels during metabolic disease can offer protection against insulin resistance and β-cell proliferation, but also lead to oxidative stress, GLP-1 receptor agonist toxic effects, and circadian rhythm disruption. Wnt/β-catenin signaling, Wnt1 inducible signaling pathway protein 1, the gut microbiome, the mechanistic target of rapamycin, AMP activated protein kinase, and silent mating type information regulation 2 homolog 1 (*S. cerevisiae*) are necessary components for FoxO biological oversight.

**Table 2:** FoxO cellular mechanistic considerations.

Chasing the FoxO in Metabolic Disorders: Novel Considerations for Oxidative Stress, Programmed Cell Death, Wnt, and the Gut Microbiome
Mammalian forkhead transcription factors of the “O” class (FoxOs) are intimately linked to multiple mechanistic pathways that impact cellular metabolism.
Cellular loss during oxidative stress is not a linear process. The detrimental effects of oxidative stress can be highly reliant upon tissue specificity, cellular energy pathways, gender, and mechanisms involving inflammation.
Autophagy can be beneficial at times, but modulation of autophagy flux levels is crucial to achieve desired biological outcomes. Heightened induction of autophagy can result in stem cell loss, oxidative stress, infection, neuronal cell injury, and circadian rhythm disruption.
In some scenarios, a critical level of FoxO activity is required for cellular viability against oxidative stress and programmed cell death. Restoration of depressed FoxO levels can be required to promote cellular protection that fosters deoxyribonucleic acid (DNA) repair mechanisms.
The silent mating type information regulation 2 homolog 1 (*S. cerevisiae*) (SIRT1) oversees the SIRT1-FoxO axis to protect against cellular injury and oxidative stress. FoxOs can be a transcriptional activator of SIRT1 and increase SIRT1 expression to limit oxidative stress, maintain metabolic homeostasis, and increase cell survival.
During metabolic disease, the Wnt/β-catenin pathway and WISP1 can control FoxOs to maintain glucose homeostasis, but entities such as WISP1 also can autoregulate their own expression, which ultimately influences cellular senescence, inflammation, and FoxO activity.
FoxOs can control the gut microbiome and gut homeostasis through the regulation of intestinal mucous secretion, which can impact short-chain fatty acids (SCFAs), producing bacteria to influence inflammatory cell activity as well as cognitive function.
GLP-1 receptor agonism prevents nuclear translocation of FoxO1 and, as a result, proliferation of pancreatic β-cells is promoted, which can improve glucose homeostasis, but careful oversight of mechanistic target of rapamycin (mTOR) and autophagy activities with GLP-1 receptor agonism and FoxOs is vital for seeking desired clinical outcomes.

## Data Availability

No new data were created in this study. Data sharing is not applicable to this article.
